# Striving towards access to essential medicines for human and animal health; a situational analysis of access to and use of antifungal medications for histoplasmosis in Ethiopia

**DOI:** 10.1371/journal.pone.0278964

**Published:** 2023-03-09

**Authors:** Eleanor Robertson, Cherinet Abera, Kelly Wood, Kabeba Deressa, Samuel Mesfin, Claire Scantlebury

**Affiliations:** 1 Department of Livestock and One Health, Institute of Institute of Infection, Veterinary and Ecological Sciences, University of Liverpool, Liverpool, United Kingdom; 2 Histoplasmosis Research Group, Care of SPANA Bishoftu, Brooke Addis Ababa, Addis Ababa, Ethiopia; 3 Independent Researcher, Addis Ababa, Ethiopia; Jaramogi Oginga Odinga University of Science and Technology, KENYA

## Abstract

Antifungal medications are vital in combatting fungal diseases that affect over a billion people annually. Antifungal medications for people and equids are scarce in Ethiopia, where lack of resources to treat fungal infection, in particular histoplasmosis, is a major health challenge. Histoplasmosis is endemic within the equine population in Ethiopia, where it is estimated that one in five horses are infected. This disease has far reaching impacts on equine welfare and the socio-economic wellbeing of families. The burden of histoplasmosis in people in Ethiopia is currently unknown, representing a blind spot in public health surveillance. Previous research has identified contact with wildlife, and domestic animal species as possible transmission pathways for histoplasmosis however, questions remain about the role of equids in human histoplasmosis. Given the close proximity of people and animals in this setting, the high level of endemic disease among equids, and the common sources of anti-fungals in Ethiopia, our study adopted a One-Health approach to examine how systemic issues affect access to, and use of antifungals to treat histoplasmosis among people and equids. A qualitative study was conducted in 6 urban regions of Oromia, Ethiopia in December 2018, incorporating semi-structured face-to-face interviews and focus group discussions. Twenty-seven individual interviews were held with doctors (n = 7), pharmacists (n = 12), veterinarians (n = 5), para-veterinarians (n = 2) and an equid owner (n = 1). Eleven focus groups were conducted with equid owners (n = 42), 3 with veterinarians (n = 6), 1 with para-veterinarians (n = 2) and 1 with pharmacists (n = 2). Transcripts were analysed using thematic analysis, and dimensions of key themes conceptualised and compared. Two overarching themes namely, ‘Structural’, and ‘Human factors’, summarised the main limitations to access to antifungal medications. ‘Structural factors’ included the national reliance on importation of medicines or pharmaceutical ingredients, inaccurate demand forecasting due to poor recording of the shortfall within the pharmaceutical supply chain, deficiencies in diagnostic capacity for fungal disease and, a healthcare system funded with a significant component of out-of-pocket expenditure. ‘Human factors’ that influenced access to antifungals included the perception of the expense of antifungals compared with competing needs such as food and education, the social stigma attached to histoplasmosis that could lead to delays in treatment seeking and, readily available home remedies or alternative treatment options. Furthermore, it was reported that trust in healthcare and veterinary provisions was undermined by a perceived lack of efficacious medications. Access to antifungals remains an urgent public health and animal welfare concern in Ethiopia. Key points within the supply and distribution chain that affect access to anti-fungals are identified, and policies that facilitate anti-fungal procurement and distribution should be reviewed. This paper highlights the structural, socio-economic and cultural factors influencing the management of infection with histoplasmosis, including how it is understood, identified and treated. This study identifies areas where further cross-sectorial work is needed to address these factors to improve disease control and clinical outcomes observed in human and animal histoplasmosis within Ethiopia.

## Introduction

Health is a recognised human right within international law and is dependent upon access to healthcare and essential medicines as outlined in the UN Sustainable Development Goals (SDGs) [1–3]. However, 30% of the world’s population lack regular access to quality-assured, affordable, essential medicines [4, 5].

Fungal infections are a major cause of human morbidity and mortality, killing more than 1.5 million people annually and affecting over a billion people worldwide [6]. This represents a similar number of deaths as those caused by AIDS and tuberculosis (TB), and over three times as many as those caused by malaria every year [6–8]. This significant health burden can be attributed to poor recognition of fungal diseases, limited access to essential antifungal medications and ineffective alternatives [9–11]. Key barriers to access have been identified such as; inefficient global medication distribution systems, prohibitive drug expense and insufficient research into fungal conditions suffered primarily by those in low and middle-income countries (LMICs) [1].

Histoplasmosis is a fungal infection caused by *Histoplasma capsulatum var*. *capsulatum* that can affect people and horses. *Histoplasma var*. *farciminosum* (HCF) is thought to be an equine specific variant and is prevalent in Ethiopia. In people, histoplasmosis has been estimated to be responsible for over 80,000 deaths globally, despite being associated with a relatively low mortality if appropriate treatment is used [9]. Histoplasmosis is a leading cause of co-infection among immunocompromised populations [12–16], and its prevalence is thought to have increased significantly in Africa alongside the HIV/AIDs epidemic [12, 16]. However current prevalence estimates are lacking, largely due to a lack of diagnostic capacity, and could be complicated by its similar clinical appearance with TB. It is acknowledged that co-infection with TB may be occurring more frequently than is recognised, and mis-diagnosis is possible in TB smear negative patients, who may not be followed up for investigation of fungal infection [15, 16]. In people, the disease has multiple manifestations with consequences ranging from no symptoms to death [17]. Acute or chronic pulmonary disease is considered the hallmark of histoplasmosis human infection, with the disseminated form of disease affecting multiple organs including the spleen, liver, skin and lungs. The burden of histoplasmosis in people in Ethiopia is currently unknown, representing a blind spot in public health surveillance. Previous research has identified contact with wildlife, and domestic animal species as possible transmission pathways for histoplasmosis however, questions remain about the role of equids in human histoplasmosis.

Histoplasmosis caused by *H*. *capsulatum var*. *farciminosum* is an endemic disease of major socio-economic importance and impacts welfare of equids in sub-Saharan Africa [18]. In Ethiopia, on average 20% of equids are estimated to be infected with histoplasmosis, however prevalence varies significantly by region [19–21]. Ethiopia has the largest equine population in Africa, the majority of which are working animals. Horses are relied upon as a primary source of income, a means of accessing markets and water sources, draught power, an affordable means of transport, social capital and financial stability [22]. When a debilitating disease such as histoplasmosis affects the ability of an equid to work, the impact on animal welfare and human livelihoods is extensive [22, 23]. It has been reported that owning an animal infected with histoplasmosis reduces the daily income potential of a family [23]. The effect of declining body condition and health of the horse results in less productive working hours and, customers avoiding horses with lesions [24]. Further losses attributable to the disease include, reduced working power, treatment costs and mortality and have been estimated at an average of 8447 Ethiopian Birr (ETB) per cart-horse owner [23].

In horses, the disease is described in four forms: cutaneous, ocular, respiratory and asymptomatic. In Ethiopia, Equine histoplasmosis is untreatable in advanced disease with currently available antifungals, and ultimately results in euthanasia or abandonment of the animal.

**Fig 1:**
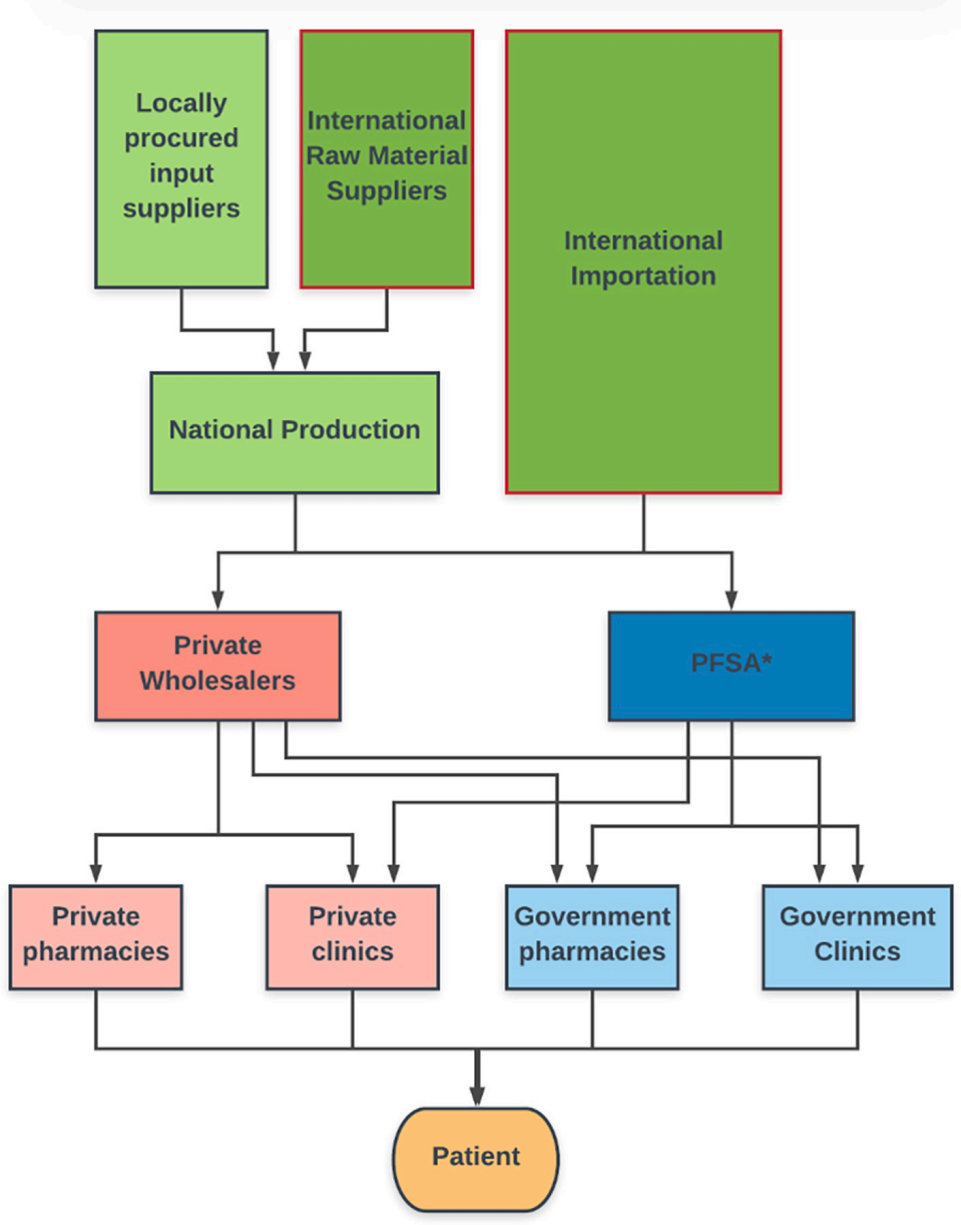
Schematic demonstrating antifungal medication procurement procedure in Ethiopia. Triangulated with the National strategy and plan of action for pharmaceutical manufacturing development in Ethiopia (2015–2025) [25] and the World Bank Health Extension Program in Ethiopia [26]. *Pharmaceutical Fund Supply Agency.

The healthcare system in Ethiopia includes government and private clinics, and both the medical and veterinary sectors acquire pharmaceuticals through similar routes. Fig 1 describes the routes of importation, acquisition and distribution of anti-fungals in Ethiopia.

Pharmaceuticals imported into Ethiopia must be registered with the Ethiopian government and are specified in the ‘List of Medicines for Ethiopia’ published by the Food, Medicine and Healthcare Administrations and Control Authority of Ethiopia (FMHACA) [27] which includes amphotericin B, fluconazole and ketoconazole.

A mainstay treatment of choice for human histoplasmosis is itraconazole [28], however this is not listed among the list of medicines for Ethiopia, and is not available in country [27, 29]. Of the anti-fungals listed, amphotericin B and fluconazole have been reported as having some efficacy against human histoplasmosis [30, 31] but have been found to be less efficacious than itraconazole [32], and further research has been recommended to inform treatment protocols for histoplasmosis in people [31].

Among the veterinary sector, government run clinics provide veterinary services for equid owners, and source their pharmaceuticals through the same routes as human hospitals / clinics. Anti-fungals are not available for equids among the government sector. There are currently a number of NGO’s working in Ethiopia including SPANA and Brooke, both of which are independent international NGO’s (INGO’s). Although some of their medicines are sourced from within national supply chains, many equine pharmaceuticals are not available, and are sourced externally and shipped independently to the charitable clinics providing services to equid owners.

Amphotericin B, the antifungal medication of choice for treating equine histoplasmosis [33], is currently unavailable for equids in Ethiopia. Furthermore, quantities required to treat horses are cost prohibitive and lack clinical trials to inform dosing regimens and efficacy. The only anti-fungal preparation that is available is potassium iodide, which is sourced independently via an INGO, however, cost, shipping and regulations make it difficult to provide a sustained supply.

Histoplasmosis and its treatment remain under-researched. Little is known about the socio-economic and cultural drivers around managing histoplasmosis in Ethiopia. Given the close proximity of people and animals in this setting, the high level of endemic disease among equids, and the common sources of anti-fungals in Ethiopia, our study adopted a One-Health approach to examine how systemic issues affect access to, and use of antifungals among people and equids.

## Materials and method

**Fig 2:**
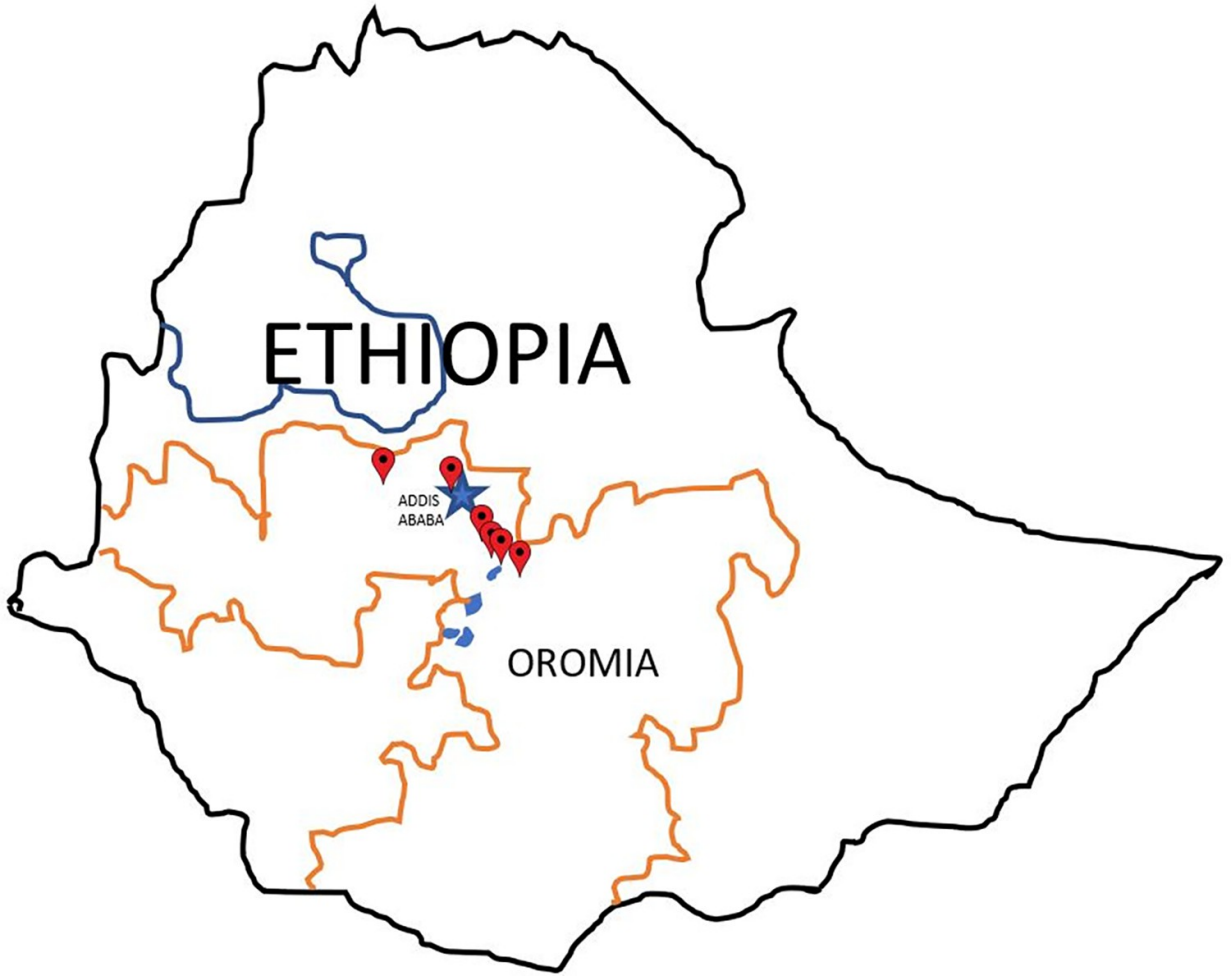
Locations of 6 urban study-sites in Ethiopia. Interviews and Focus Group discussions were held in each of these areas (S1 Table).

A qualitative study was carried out in 6 urban regions of Oromia, Ethiopia in December 2018 to explore factors involved in access to and use of antifungals (Fig 2). The study adopted a basic social constructivist approach [34, 35] and involved in depth face-to-face interviews, and focus group discussions among stakeholders involved in prescribing and consumption of antifungals.

Participants were invited to the study via a veterinary international non-governmental organisation (INGO), (SPANA Ethiopia), medical practice managers or pharmacy owners. The research team first sought permission in each of the 6 study regions from the head of the veterinary satellite clinic, with a letter supported by the College of Veterinary Medicine and Agriculture. Permission was also sought from the head of each institution attended prior to selecting medical and pharmacist participants. All participants were briefed about the purposes of the study in their primary language (usually Oromic or Amharic), and asked to provide written voluntary informed consent. No personally identifiable information was recorded. Ethical approval was obtained from the University of Liverpool (VREC717) and was reviewed by the College of Veterinary Medicine and Agriculture Research Ethics Board, Addis Ababa University (review reference D/399/2018). Additional information regarding the ethical, cultural, and scientific considerations specific to inclusivity in global research is included in the (S1 Checklist).

The six study regions were selected based on their high population of equids and the presence of veterinary satellite clinics of the INGO with whom the study team were working.

Twenty-seven individual interviews were held with doctors (n = 7), pharmacists (n = 12), veterinarians (v = 5) and veterinary paraprofessionals (n = 2) and an equid owner (n = 1). Sixteen focus groups were conducted: eleven with equid owners (n = 42, with an average of four participants per discussion), three with veterinarians (n = 6), one with para-veterinarians (n = 2) and one with pharmacists (n = 2).

Each of the six INGO satellite clinics were visited once during the data collection period, and participants were invited amongst those attending clinics on that day. Equid owners were purposively selected to include those with a range of years of experience of keeping horses. Due to the attending population, it was mainly male participants that were included, and this is consistent with the veterinary clinic attending population. Equid owners (n = 43) had kept horses from between eight months and seven years (see S1 Table for further details of participant demographics). As far as possible, focus group discussions were formed to include those with similar age to encourage all to participate freely.

Veterinary clinicians were invited to participate from each of the six INGO satellite clinics, where both INGO and government veterinarians were present. We aimed to interview veterinarians with a range of experience, our sample represented a range of career stages including newly qualified (n = 3), and graduates (n = 8) ranging from two to 30 years’ experience (median five years), and veterinary paraprofessionals (n = 4). Focus group discussions were carried out among those with similar professional standing in order to encourage all to participate freely.

During the equid owner focus group discussions, we asked participants where they attended for medical and pharmaceutical treatments in the local vicinity. The research team then reached out to the directors / managers of these institutions to seek permission to invite doctors and pharmacists to take part in in-depth interviews. The directors / managers directed the research team to which professional would be available on that day to volunteer for the study. Doctors were recruited from local primary community health posts (n = 2), general practitioner clinics (n = 2) and specialist dermatology clinics within tertiary referral hospitals (n = 3). Pharmacists were recruited from a mix of private (n = 4), government (n = 8) and animal pharmacies (n = 2).

Semi-structured interviews explored a range of topics of interest that were pre-determined by the research team, based on experience of working within the veterinary field and previous research within this context (S1 File). Key areas that were explored included: perceptions of fungal disease, perceptions of antifungal treatment, treatment seeking behaviours, practices surrounding medication procurement and, perception of zoonotic risk. Case photographs were shown to equid owners, veterinary surgeons and pharmacists to provide examples and facilitate discussion. Open-ended questions were asked to initiate discussion, followed by exploration of the respondent’s views, experiences, and knowledge using open probing and extension questions. A case radiograph [36] and vignette were presented to doctors to explore approaches to diagnosis (S2 File).

All interviews and focus group discussions were recorded with a Dictaphone, and hand-written memos were made following reflection and discussion with the data collection team. Audio recordings were translated from Oromic or Amharic into English and transcribed verbatim by an Ethiopian national (SM).

Transcripts were analysed using a Thematic analysis approach, whereby data was coded into categories to aid in identification of patterns and the development of conceptual relationships across the different transcripts [37]. ‘NVIVO’ software was used to assist in cataloguing and organizing themes, while all coding was conducted manually. At first, line-by-line open coding served to label and classify topic themes and main concepts. An iterative process of review of these codes and their relationships to other data, led to themes being grouped into overarching concepts, and the links between these concepts were further explored through axial coding [38]. Initially, transcripts were analysed within stakeholder groups, then, as axial codes developed, comparisons and reflections were enabled between groups (e.g., between veterinarians and pharmacists, and veterinarians and doctors).

Data saturation was achieved in the equid owner and veterinary participant groups, indicated by no new concepts or ideas arising within the interviews, a further two interviews beyond saturation were conducted to confirm this within these groups. However, the pharmacists and doctors continued to contribute new details to the concepts throughout the data collection.

## Findings

Overall, two main areas, namely, structural, and human factors were found to be contributing to the challenge of effectively managing fungal infections within the human and animal populations.

### Structural factors

Structural factors are the systems and institutions that exist within a context. Three key structural factors were identified as impacting the use of and access to antifungals in this setting. These were national and local pharmaceutical provision, the supply of veterinary treatment and, the capacity for diagnosis of histoplasmosis.

#### National and local pharmaceutical provision

Pathways for procuring antifungal medications were highly complex and included multiple steps. Bureaucratic and logistical barriers were identified at international, national and local levels. The health system in Ethiopia spans both government and private sectors and issues relating to sourcing and availability within each of these were inter-dependent (Fig 1).

At a national level, four key factors were identified as explanation for the poor availability of antifungal medication. These were: reliance on international importation, the low priority of antifungal importation, factors affecting domestic production and, systems for procuring antifungals.

The majority of these antifungals were imported however, the fluctuation of the Ethiopian Birr (ETB) made this route vulnerable within the international market. *‘For nine months*, *because of the [strength of the] dollar*, *all drugs are not available in Ethiopia*. *Import and export are closed because of this*’ (Interview 20; private pharmacist).

Participants voiced frustrations with the low political priority of antifungal drugs, that resulted in insufficient stocks to sufficiently meet demands of the population, as one pharmacist shared, ‘*You have to tell them*! *Please*! *Please seriously invest on antifungal drugs*. *Since there are poor people living with the disease*, *they are extremely poor and can’t afford the drug*, *they live with the disease*’ (Interview 6; private pharmacist).

Ethiopian produced pharmaceuticals could be one solution to the unreliable international importation of medication, although currently production is insufficient to meet demand. A challenging requisite for local manufacture of antifungals, is the importation of raw materials that are subject to the same difficulties as medication importation. This is a hinderance to the growth and development of local production, as it means national production can be easily outcompeted by larger international pharmaceutical companies who can produce in a more sustained, cost-effective manner. Ethiopian produced drugs were sometimes perceived to be of lower quality by the public, and this was suggested to affect client compliance. Pharmacists reported that clients preferred the imported European brands, followed by either Chinese or Indian products over the Ethiopian made brands. *‘The patients have no confidence (in the domestic products) and they complain most of the time*. *They prefer imported drugs*. *They hate the appearance*, *and the taste is better from the imported’* (Interview 36; public pharmacist).

The local procurement procedure for clinics and pharmacies was complex and identified as a significant barrier to the supply of antifungals. The government-operated Pharmaceutical Fund Supply Agency (PFSA) was the key distributor of antifungal medications to government pharmacies and both private and public clinics (Fig 1).

PFSA was described to be preferred over private vendors as it was cheaper, but the supply was reported as intermittent. This supply challenge was four-fold. Firstly, individual pharmacists were required to travel to the distribution centre in Addis Ababa, which is associated with significant costs if they are forced to wait for unavailable medications. *‘They will say it’s out of stock and when you go back to [PFSA] they say wait it will be coming*, *but you can’t stay and wait there because there are a lot of costs*, *for transportation and accommodation*, *the office that you are working at may complain about you…*’ (Interview 28; private pharmacist).

Secondly, participants expressed concern of an apparent lack of impact of feedback on product demand, despite available reporting mechanisms. One government pharmacist shared, ‘*Yes*, *there is (auditing of our demand)*. *The storemen fill out the paper*, *the consumption ratios*. *Every quarter of the year we send that to the government*, *they see the demand*, *but I don’t think they can do anything about it*, *at least that is my guess’* (Interview 39; public pharmacist). This highlights a significant challenge within the system to accurately record and respond to the need for antifungals.

Thirdly, the unreliable provision of medications meant that often government pharmacies and clinics were forced to purchase up to 50% of stock from the private market instead of PFSA, pushing prices up for the consumer. The price increase, sometimes over four times the public sector price, was associated with low patient access and compliance. This was a source of dissatisfaction as, *‘if the private [clinics] have it*, *the government should be able to get it too*. *The cost would be cheaper*, *and the community can afford it*’ (Interview 28; private pharmacist).

Fourthly, government pharmacists were obliged to demonstrate an inability to access medications from PFSA and required to apply for permission before purchasing goods from the private market. This made the procurement procedure cumbersome. One pharmacist shared that ‘*If [we] can’t get through public*, *[government channels]*, *there is a lot of work involved*, *you have to convince [our regulators PFSA] to allow us to buy from the private*, *then you have to search*, *you have to find the lowest price… This takes months*, *two to three months*. *It takes time’* (Interview 39; public pharmacist).

#### Veterinary pharmaceutical supply

In Ethiopia, veterinary pharmaceuticals are regulated independently from human medications by The Ministry of Agriculture and Rural Development. Formal treatments for histoplasmosis in equids were listed by veterinary professionals as topical tincture of iodine, oral or intravenous iodide salts and adjunctive antimicrobial therapy.

Veterinary healthcare was divided between government and international NGO clinics. Among our horse owner and veterinary respondents, antifungal drugs were reported never to be available through the government veterinary system, and topical iodine was rarely purchased through human pharmacies.

Within the study sites overseen by government veterinary clinics, iodide salts were never present and, tincture of iodine was often in short supply. It was believed that PFSA would be unwilling to sell pharmaceuticals to the veterinary industry, possibly due to the prioritisation of human health in the face of a medication shortage.

The NGO clinic had access to international donations that enabled them to purchase and import potassium iodide salts, but their availability was intermittent due to interruptions in the supply chain. Without the support of the NGO clinic, it was thought that equid owners would be unable to buy this product as, ‘*It will cost around 2000 birr (approx*. *38 USD Nov 2022) … the owners can’t afford that*. *2000 birr is nearly one third of the horse cost*’ (Interview 16; Vet) and was not stocked by pharmacists. The high cost was associated with the sourcing, transportation and importation of the product and tight shipping regulations created additional complexity. The inconsistent availability of potassium Iodide within the NGO clinic created tensions affecting client compliance as well as frustration among veterinarians, … ‘*[Owners] know about the iodine tincture and the potassium [iodide]*. *They too know… which one is better [namely the potassium iodide] … But it’s often not here… If the drug is not available*, *they will choose to leave*, *and they will not choose to use the iodine tincture’* (Interview 15; vet assistant).

In the absence of antifungal pharmaceuticals, equid owners frequently sought alternative treatments. These included herbal dietary supplements, topical pastes or injections, firing of the lymphatic vessels with hot metal, faith-based rituals such as blessed water and the topical application of fly repellent and vehicle grease or caustic products such as battery acid. One equid owner explained, *‘When our horse suffers*, *we will bring here [to clinic] and there is no effective treatment*, *so we will use the traditional treatment*. *[We] gather leaves from the trees and dry it and make it in powder form and make an ointment on the horse*’ (Interview 29; equid owner).

Traditional treatments were perceived to be readably available, cheaper and believed to have similar, or better clinical outcomes than the pharmaceutical options. Furthermore, there was widespread belief that the disease was most likely fatal despite treatment, therefore, cheap and rapidly acting therapies were sought to avoid money and time being wasted on an animal that could not work.

In particular, firing was identified by owners as a regularly used and effective method to stop the spread of disease, ‘*First we try by ourselves*, *we burn*… *Once we burn that area it stops there and doesn’t spread*’ (Interview 6; equid owner). However, this method is strongly rejected by charitable veterinary NGOs owing to its high level of pain and tissue destruction and administration without suitable analgesia or anaesthesia.

#### Diagnostic capacity for histoplasmosis infections

In equids, diagnosis of histoplasmosis was based largely on clinical presentation. Occasionally, a pus sample would be stained and examined microscopically, but this was the limit of any further diagnostic investigation. One vet said, ‘*We don’t have laboratory equipment*, *reagent or technician like that to confirm such like disease*. *We just diagnose by ourselves’* (Interview 24; vet assistant). This lack of diagnostic capacity, may be resulting in misdiagnosis, underreporting and possible misallocation of scarce treatment resources.

Diagnostic pathways were explored within the medical interviews with referral to the case vignette of a chest radiograph in an HIV positive patient. Participants recognised that immunocompromised individuals have an increased susceptibility to histoplasmosis and other fungal infections. However, doctors highlighted that HIV and malnourishment also predispose patients to other infectious agents such as TB. In many instances, doctors acknowledged that TB would be the primary differential diagnosis, with limited further diagnostic investigation. Furthermore, diagnosis was often assumed due to the high prevalence of TB in the region, ‘… *TB [is] probable… it is common in HIV positive individuals*. *It will be in the list of our differentials*’ (Doctor interview 5) and … ‘*[Histoplasmosis] resembles TB…so we consider TB*. *Even if it’s negative result*, *since it’s epidemiological*, *we consider that*’ (Interview 4; tertiary clinic doctor).

The overlapping symptoms and similar radiographic findings with both TB and histoplasmosis complicated clinical decision making, further hindered by limited diagnostic capacity, including a lack of time and resources in tertiary hospitals, and scarcity of diagnostic equipment in primary clinics.

Misdiagnosis was deemed plausible … ‘*Sometimes you can miss this*, *because of the load of the work*. *All doctors can’t think*, *‘but something is remaining*?*’ If the case is not improved [with treatment] you will test again…*. *reinvestigate’* (Interview 2; tertiary clinic doctor). As such, histoplasmosis may be being misdiagnosed, underreported, and mis-treated due to the similar clinical presentation and high TB prevalence in the area.

### Human factors

Human factors were identified as those concerned with the interactions between humans and their responses to existing institutions and systems within which antifungal medications are used. In this study these were, the perception of antifungal treatment, trust in health and veterinary care, the impact of fungal disease and, biosecurity and disease prevention.

#### Perception of antifungal treatment

Across both human and veterinary healthcare systems, a number of factors shaped people’s perceptions of antifungal treatment and influenced decision making. These included, the perceptions of accessibility, in terms of physical access and cost, time commitment required, and safety. Perceived efficacy also played an important role in the veterinary treatment seeking by equid owners.

Doctors’ prescribing practices were influenced by the perceived poor availability of medication. This seemed to originate from experience of patients returning to clinicians with requests for different prescriptions, when the original prescribed medication could not be obtained through pharmacies. … ‘*Choice is based on textbooks and clinical experience and availability*. *Also*, *affordability*. *Very often [we] have challenges with availability- [this] really effects the treatment choice*’ (Interview 3; tertiary clinic doctor). This compounded the issue of auditing and perceived need for antifungals as; if a medication is not perceived as available, and therefore not prescribed or requested, the recorded demand will decrease and contribute to minimal production, or reduced importation.

Doctors and pharmacists emphasized cost as a key factor in the inaccessibility of antifungals. Costs were often high due to the long duration of treatment, common reoccurrence of fungal infections and, the necessity to access drugs from the private sector. This either limited the initiation of any treatment or, resulted in a sup-optimal treatment regime. … ‘*Around 50% of patients that are prescribed antifungals end up with the medication–mostly due to price… the duration is greater than one month and so the cost adds up*’ (Interview 9; government GP doctor). It was reported that patients may start the treatment but run out of the drug and be unable to find it again, thus not completing the course. Doctors and pharmacists were most frustrated that antifungal medication could not be reliably provided for HIV positive patients, for whom they viewed it as essential to receive timely treatment.

Patient perception of drug safety created hesitancy in the use of antifungal drugs. Participants commonly described side effects and discussed how this affected patient compliance, primary concerns included, but were not limited to, gastro-intestinal upset or liver toxicity.

Among owners whose animals were affected by the disease there is high demand for anti-fungal treatments that are effective and affordable. However, the use of antifungal medications was reportedly rare within the veterinary profession owing to a lack of available medicines. Instead, veterinarians and equid owners aimed to disinfect the wounds and treat secondary bacterial infections. Equid owners observed the limited success of these supportive treatments, which in turn discouraged them seeking veterinary assistance. During a group discussion a participant shared, ‘*There is no effective medication and once they are affected with this*, *they cannot recover*…*once it is caught*, *the only chance they have is do die’* (Interview 31; Equid Owner). This limited access to curative treatment often led to equid owners being forced to consider euthanasia or abandonment of horses that were severely infected and no longer able to work as there was little hope of recovery.

#### Trust in health and veterinary sector

The lack of effective treatment distressed both the veterinarians and the horse owners. One vet shared, ‘*Everyday… [I am]*…*seeing animals suffering*, *I don’t have options… it is very serious’ (*Interview 16; vet*)*. Participants explained that the equine veterinary sector was competing for resources with both the prioritized meat producing farm species and the human health sector…*‘it is a matter of budget allocation…we share it with other farm animals*, *so they focus on other drugs rather than this…*The *government is not giving attention to the veterinary services; they focus more on human [health services]*… *Look at my compound and my room … we have nothing*. *We simply sit and have paper*. *While we are here*, *so close to Addis*. *Imagine what people in rural areas have—nothing*, *the government needs to do more’* (Interview 40; vet).

There was concern about the reputation of the profession, and frustration and demotivation due to the lack of resources. It was feared that, with lack of effective medication limiting veterinary capacity, trust in the profession would be harmed… *‘[Veterinarians] need to have access to more specific drugs in order to improve their own confidence in the treatment and the community’s faith in the veterinary profession’* (Interview 24; vet assistant).

This concern was echoed by equid owners who frequently discussed alternative treatment options to formal veterinary treatments. They described the alternatives to have higher success rates, faster recovery times, or simply felt that it was worth trying everything, as nothing was considered to hold much promise. Traditional therapies were often provided by trusted members of the community or by the owners themselves which greatly increased their accessibility compared to formal veterinary treatment.

**Fig 3:**
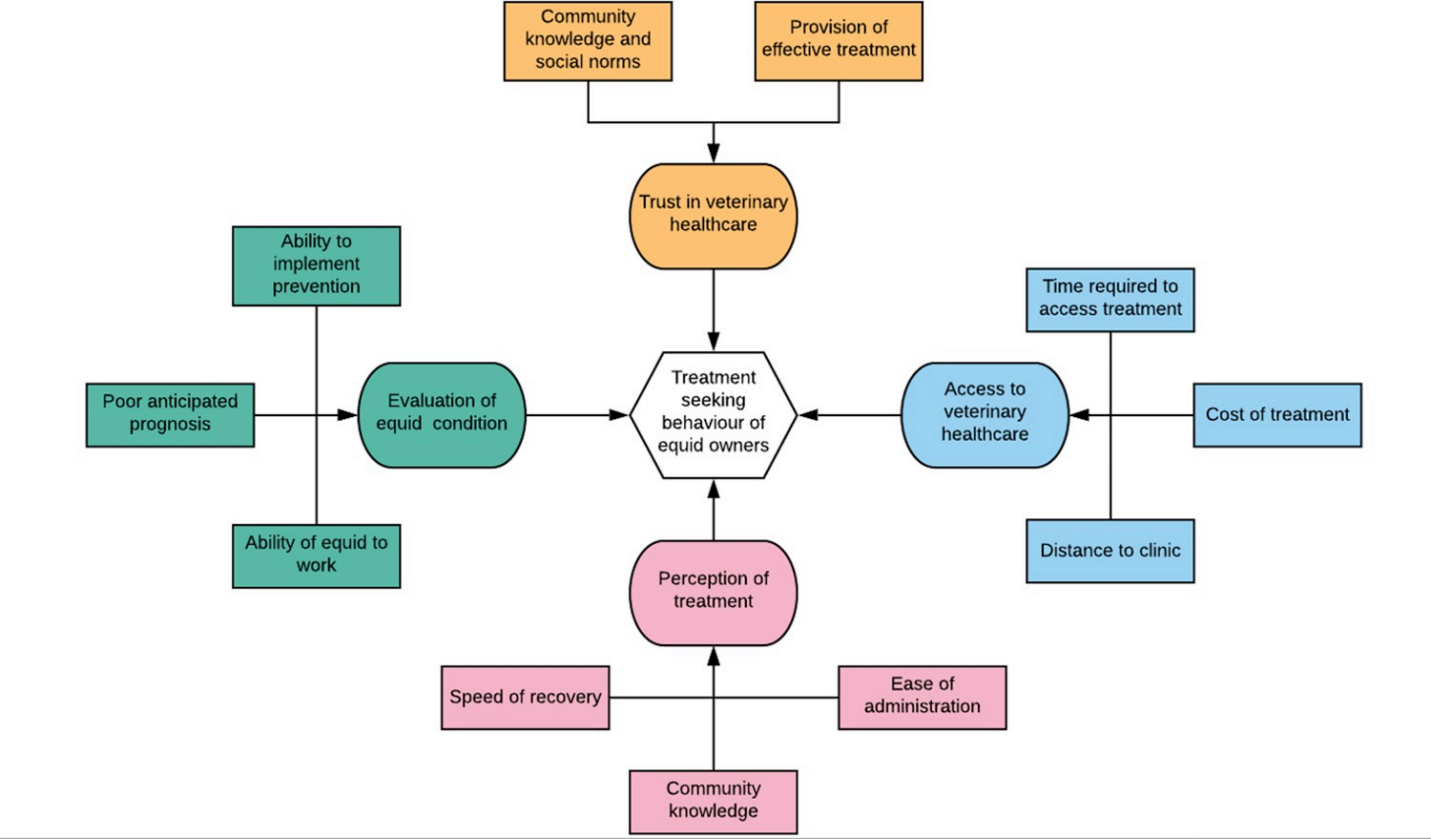
Factors contributing to the veterinary treatment choices of equid owners in Ethiopia.

Many equine owners are farmers who must travel a long way to access veterinary clinics. Equid owners reported that often there appeared to be no benefit in travelling longer distances to seek veterinary advice when no effective treatment could be secured (Fig 3). ‘*most of our clients complain as it is difficult…*. *most of our farmers come from a very far area and most of these horses are used for work*. *They say*, *‘we are stopping our work and we have come a long way to do this’*. *Some people complete [the course of treatment]*, *and others maybe stop in the middle [of treatment]—because of the obstacles in the way’* (Interview 32; vet). This highlighted the low level of owner confidence in veterinary provision available for this disease, a lack of motivation by owners to seek treatment if it is not easily accessible and represents a challenge for disease control.

In contrast, the same equid owners, when asked about matters of human health, reported they would almost always go to the local health centre rather than a community member to seek advice or treatment. This decision appeared to be founded upon a difference in the level of trust in the available treatment. Further, the lack of efficacious treatment at veterinary clinics enhanced owners’ perceptions of an increased risk of disease transmission from infected horses attending the clinic.

#### Impact of histoplasmosis fungal disease

Veterinarians and equid owners emphasized the devastating effect that equine histoplasmosis has on human lives as well as the direct animal welfare impacts. Losses due to histoplasmosis generated significant human burdens including economic losses, both in terms of capital and income, social impacts, such as isolation from fellow equid owners and, the emotional impact of losing an animal.… ‘*They are our means of life…when we lose them*, *our lives are devastated*. *Our lives depend on them… When our horse is affected by this dangerous disease*, *we are getting into some severe problems’* (Interview 37; horse owner). Another owner described the emotional impacts of losing an animal. ‘*I really love my horses… the only difference that they have with my child is the place where they sleep*’ (Interview 37; horse owner). The owners of equids with histoplasmosis lesions reported feeling stigmatized due to the association of fungal infections with poor management and hygiene, similar to attitudes reported about human fungal infections. Those with infected horses were assumed to care inadequately for their animal and were shamed for that, and this impacted on their ability to generate an income. In advanced disease the horse smells bad and clients would avoid employing their cart. Additionally, if an equid has extensive histoplasmosis lesions, often other drivers would isolate the horse and their owner to prevent spread. … ‘*Even friends will isolate the people and the horses*, *will no longer feed or drink together because they want to protect the healthy one’* (Interview 12; horse owner). The social and physical isolation experienced by the owners of histoplasmosis infected horses could create a reluctance to visit clinics on histoplasmosis specific treatment days for fear of being stigmatised.

Doctors also reported the role of stigma in patient treatment seeking behaviours. In people, it was primarily the association of HIV positive status and fungal infections that was of primary concern, as fungal lesions were physical indicators that could belie the status of the individual … *‘if they have the HIV they will be socially isolated*. *There is stigma…if it’s known that they have the HIV in the society*’ (Interview 34; government GP doctor). This had an impact on people coming forward for treatment.

#### Biosecurity and disease prevention

The limitations of available treatments highlighted the importance of biosecurity measures to reduce transmission. Many equid owners described barriers to them seeking treatment such as, the chronic nature of disease creating an illusion of no rush to seek treatment, and a reduced motivation to seek treatment due to ineffective and unaffordable treatment options. Improved biosecurity measures act to reduce infection rates and reduce the burden of requirement for anti-fungal treatments.

Ideally horses with histoplasmosis lesions should either be isolated and treated until clinical resolution, or euthanized. These biosecurity measures faced implementation challenges such as, shared grazing pastures reduced opportunities for isolation, and owners were under financial pressure to keep a horse in work. Income generated by the animal supported family essentials such as food, medicines and educational fees. Consequently, equids continued to be worked and were abandoned at the point where they had not responded to treatment and could no longer work. Abandonment was often seen as the only option because owners could not afford to feed and house an animal not providing an income, they recognized the risk to other equids within the household, and this tied in with the concept of not deciding to euthanize to give the horse any possible chance to live.

Euthanasia was not favoured due to a combination of cultural, ethical, financial and logistical considerations. The value of equine life was considered highly among owners and linked with a respect for the human horse relationship *‘I will not let the horse that served me for years die [by euthanasia]*. *I will help him through the medication until I can*, *but I will not let him die that way’* (Interview 31; Equid Owner), as well as religious beliefs, ‘*No one will do that*. *We want them to be dead on the day God has decided… God created them so why do we kill them*?’ (Interview 38; Equid Owner). There was further reluctance to euthanize if there was any chance of recovery, due to the value of the horse as a financial asset ‘*We have got income by the horse…we will not allow our animals to be killed because it might get better*.’ (Interview 22; Equid Owner). Veterinarians also viewed euthanasia as a challenge due to resource and logistical constraints, one vet shared, ‘*We don’t have those drugs [to euthanise a horse]*. *We don’t have those facilities*. *After the euthanasia we need transportation to discard the carcass and we don’t have that*.’ (Interview 32; Vet).

## Discussion

Overall, anti-fungal medications were found to be difficult to source and prohibitively costly to patients and equid owners. The Food, Medicine and Healthcare Administration and Control Authority of Ethiopia has adopted the World Health Organisations (WHO’s) essential medicine list recommendations, and published the ‘List of Essential Medicines for Ethiopia’ [27]. This list includes amphotericin B, fluconazole, and ketoconazole. However, Suleman *et* al., (2016) [39] found that although adequate pharmaceutical regulation exists in Ethiopia, the existence of policy alone was insufficient to ensure adequate supplies of these medicines without adequate political and resource commitment [39]. This was highlighted by a reported lack of, or only intermittent access to these listed anti-fungal medications. Indeed, participants of this study identified that amphotericin B, fluconazole and ketoconazole were only intermittently available with no access to Flucytosine. Through limited stocks and unaffordable pricing, access to these essential medicines remains a serious challenge to human and animal health [40].

The majority (65%) of pharmaceuticals are imported into Ethiopia and are purchased by the public sector (PFSA), with the rest being private, non-governmental or international organizations [41]. The open tender process used by PFSA is lengthy and complicates long term relationships with providers. This creates barriers to sustainable supply and contributes to the difficulties pharmacists and clinicians report here in providing these treatments to patients.

Local pharmaceutical production represents less than 20% of the market, and is reliant on importation of the vast majority of constituents (up to 98%) [41]. Local production is encouraged by a) pharmaceutical producing companies being made exempt of paying duty tax on 80% of their imports [41] and b) PFSA giving preferential treatment to local manufacturers [41]. These initiatives aim to promote sustainable medicine use, and similar policies have been adopted in other countries on the continent [42, 43]. However, there are ongoing challenges with local production. Pharmaceutical production is thought to be constrained by the high technical standards and strict quality controls needed to ensure safe product [41]. These quality assurance processes are compounded by excessive competition with international low-cost, largescale pharmaceutical production. This has led to Ethiopian based firms struggling to finance production and meet demand [41]. Furthermore, these findings suggest that, even if local production of antifungals could increase in efficiency, work would be needed to boost their reputation for quality among the public.

The combination of prolonged treatment courses and poor availability of publicly sourced medication results in an unaffordable cost of treatment for both humans and working equids. Prohibitive cost of treatment often leads to inferior management of fungal infections impacting on patient outcomes, while interrupted or uncompleted treatments, can increase the development of anti-fungal resistance, complicating the clinical picture and driving evolution of fungal pathogens [44]. At the current time, the anti-fungal resistance profiles of Histoplasma strains in people and animals in Ethiopia is unknown, and is a critical element to understand when aiming to improve access to anti-fungal treatment.

Ethiopia is the fastest growing economy in the region [45] and has made significant progress in improving health outcomes since the introduction of the millennium development goals [46]. However, public funding per capita spent on health is among the lowest in Africa, and the majority of payments into the healthcare system are out-of-pocket payments by patients or international donors [46]. Of the government funds that are available, there is little flexibility in their allocation [47]. A fee waiver system developed by the Ethiopian government that aims to protect the most vulnerable from poverty induced by medical expenses is a great initiative. However, it has been suggested that improvements are required in the accurate identification of the appropriate beneficiaries [46]. Reduced or free medical services provided are primarily limited to pre or post-natal care, TB and HIV related testing and treatment [46]. This focus on high profile diseases such as TB and HIV have meant that the infrastructure and international funding has often been disease-specific, often overlooking fungal disease. However, fungal infections such as histoplasmosis are important contributors to the morbidity and mortality of these priority diseases [48].

The cost of antifungals is largely unaffordable for working equid owners. It is estimated that treatment of histoplasmosis cases with imported anti-fungals cost over a third of the market price of the horse in 2010 [49], with costs increasing significantly since then. This high price is unattainable for an animal kept as a means of income, and therefore seeking alternative remedies, traditional medicine, abandonment or euthanasia become the only viable options for these owners.

Informal treatments for histoplasmosis are widely used among working equid communities. Commonly, herbal based remedies and ‘firing’ were applied, both of which have been met with controversy over concerns for animal welfare. The Royal College of Veterinary Surgeons (RCVS) in the UK, banned the use of firing in veterinary practice in the early 1990’s [50]. However, in the absence of effective medication in Ethiopia, owners often viewed firing as an effective, quick and low-cost alternative. Without research regarding the practice’s efficacy or welfare implications, it is difficult to justify this technique, and there is concern around firing without the use of appropriate anaesthesia or analgesia, or expert knowledge of impact on regional anatomy. The condemnation of the practice of firing by equine charities and the veterinary profession has resulted in owners hiding the practice. Previous efforts to discourage the practice in Ethiopia are proving insufficient as, although they carry important messages regarding welfare, there is often no substitute available to equid owners who are attempting to eliminate the burden of the disease.

Herbal, or plant-based treatments were popular. Although evidence is lacking for many traditional remedies, one plant, *Phytolacca dodecandra*, was demonstrated to display antifungal properties [51]. The study found that 58% of cases treated with an *n-*butanol extract from the plant responded to treatment. In this complex context of lacking conventional treatment options, economic constraints and weak perceptions of effectiveness by equid owners, the existing local knowledge and factors promoting acceptance of alternative treatments should be explored and factored into programmes aimed at disease control.

There is a clear need for co-ordination across multiple sectors to tackle this socio-economic health issue in order to improve the current landscape of anti-fungal access and use. The current lack of comprehensive understanding regarding where the impact of histoplasmosis is felt prevents a shared acknowledgment of the roles and responsibilities of each sector. The apparent lack of political priority given to fungal diseases in Ethiopia may originate from a lack of epidemiological evidence upon which to justify investment in antifungals. Further research to describe the scale and nature of impact of histoplasmosis in Ethiopia is warranted along with further elucidation of co-infection dynamics in relation to HIV and tuberculosis within the human population. The metrics used to measure the burden of histoplasmosis, should be communicable between sectors so that the true sum impact can be recognized at all levels and tackled.

Histoplasmosis has been identified as the main differential for human TB and a leading cause of secondary infection in HIV positive patients in French Guiana and many regions of Sub-Saharan Africa [16, 48]. Co-occurrence of two or more of these diseases contribute significantly to morbidity and mortality. However, in Ethiopia, histoplasmosis is underrecognized, and doctors reported treating clinical signs empirically as TB. This contributes to a cycle of misdiagnosis, underreporting, and under recognition of the disease with knock-on effects on demand and supply of anti-fungals. In equids, the common cutaneous form of histoplasmosis presents similarly to cutaneous glanders (farcy), ulcerative lymphangitis, sporotrichosis or cutaneous lymphosarcoma [18]. Clinicians and veterinarians in this study reported a lack of diagnostic testing capacity which makes misdiagnosis possible, reduces disease identification and reporting and reduces ability to advocate for access to appropriate medications. Resources for diagnostic testing is a key component in supporting clinical decision making and appropriate allocation of anti-fungal medications. Equitable access to fungal diagnostics is an important global health goal, and GAFFI (Global Action for Fungal Infection https://gaffi.org/why/diagnostic-deficiencies/) advocates for improved diagnostic capacity to support clinical decision making and resource allocation in LMIC’s.

Control of equine histoplasmosis in endemic areas is primarily achieved by correct identification of cases through robust diagnostic testings, the challenges associated with which have been highlighted, euthanasia of infected horses and, preventing transmission through improved hygiene practices [18]. In Ethiopia, the practicalities of euthanizing infected horses presents a number of challenges including, limited access to the resources required to perform euthanasia, logistical challenges of carcass disposal and biocontainment and, a lack of cultural support of the practice. It is important to consider that any interventions to introduce disease control should be designed to support people, rather than rely on human adaptation. Findings from this study suggest that to ensure sustainable progress for a control programme to tackle histoplasmosis, consideration must be given to the social and financial factors that are associated with the disease. Two key aspects that need to be addressed include the adequate incentivization of improved biosecurity, ideally incorporating mechanisms to provide compensation for loss of the animal and, constructive conversations addressing end of life care and informal treatment options.

A successful study in India demonstrated an example of a disease control program aimed at preventing Glanders, that tackled the financial loss associated with the euthanasia of a working animal. The collaboration between non-governmental organizations and policy makers worked in tandem with equid owners’ to develop the programme, and increase its integration within communities [52]. In Ethiopia, where access to veterinarians remains limited, it is essential that community level-surveillance and disease control engages with multiple sectors and includes equid owners who are the first point of disease recognition and gatekeepers to treatment seeking. Paraprofessionals, who perform essential animal health roles, outnumbered veterinarians almost 10-to-1 in 2017 [53], and could be utilised to increase the reach of such programmes and promote widespread coverage of these messages, along with other trusted community sources. These could involve pharmacists who often prescribe animal medications and community members currently involved in veterinary and informal treatments.

Based on the findings here, four key areas warrant further action. These are i), quantification of the true impact of fungal disease in order to reflect the actual need for anti-fungals, ii), addressing the insufficient access to antifungals with a focus on improving sustainability of supply and affordable pricing, iii), public health intervention planning such as educational opportunities regarding recognition and management of clinical fungal infection, and iv) the development of achievable and sustainable disease control strategies.

The limited access to essential anti-fungal medications is an important and urgent threat to both human and animal health in Ethiopia. This study has presented key challenges to the access to and use of antifungals, highlighting the role of both structural and human factors. Current demand for anti-fungal medications is masked by a number of factors including lack of diagnostic tests and laboratory expertise, under-reporting of disease, and gaps in pharmaceutical auditing and supply chains representing target areas to initiate improvement of access to anti-fungals. These context specific realities offer opportunities for public health intervention and policy, which will rely upon effective investment in the animal and human health systems alike.

## Supporting information

S1 Checklist Inclusivity in global research. (DOCX)

S1 Table Table of interview and focus group participant demographics. (DOCX)

S1 File Interview topic guides. Key questions and topic areas for equid owners, veterinary surgeons, medics and pharmacists.
(DOCX)

S2 File Case vignette for doctor interviews. (DOCX)
