# Supplementary material for: Striving towards access to essential medicines for human and animal health; a situational analysis of access to and use of antifungal medications for histoplasmosis in Ethiopia
Source: PLoS One. 2023 Mar 9;18(3):e0278964. doi: 10.1371/journal.pone.0278964 (PMC9997978; doi:10.1371/journal.pone.0278964)
Supplement: S1 Checklist — (DOCX) [file pone.0278964.s001.docx]

Inclusivity in global research

PLOS’ policy on inclusivity in global research aims to improve transparency in the reporting of research performed outside of researchers’ own country or community and ensures that PLOS publications reporting global research adhere to high standards for research ethics and authorship. Authors of relevant research articles may be asked to complete the questionnaire below, which outlines ethical, cultural, and scientific considerations specific to inclusivity in global research. This questionnaire may be requested when researchers have travelled to a different country to conduct research, if research uses samples collected in another country, research with Indigenous populations or their lands, or if research is on cultural artefacts. Researchers travelling to another country solely to use laboratory equipment will not normally be required to complete the questionnaire. However, the questionnaire can be requested at the journal’s discretion for any submission – if you have been requested to complete this questionnaire by the PLOS journal you submitted to, please do so.

Please complete the questionnaire below and include this as a Supporting Information file with your manuscript. Note that if your paper is accepted for publication, this checklist will be published with your article in the supporting information files. Please ensure that you reference the checklist in the main body of your manuscript. We suggest adding a subsection ‘Inclusivity in global research’ to your Methods section and adding the following sentence: “Additional information regarding the ethical, cultural, and scientific considerations specific to inclusivity in global research is included in the Supporting Information (SX Checklist)”

The questions have been designed to be applicable to a wide range of study types, and there are subsections for both human subjects research and non-human subjects research. If any of the questions are not relevant to your research please mark them as “N/A” as appropriate.

**Ethical considerations, permits and authorship**

*This section is applicable to all research types.*

Provide details as to who granted permissions and/or consent for the study to take place in the Methods section of your manuscript. This should include the names of **all** ethics boards, governmental organizations, community leaders or other bodies that provided approval for the study. If individuals provided approval refer to these people by their role or title but do not list their name(s).

Reported on page number: Permission for the study to take place was provided by the College of Veterinary Medicine and Agriculture, Addis Ababa University, Ethiopia and the University of Liverpool ethics committee. This is stated in the methods section in lines 106-111.

If there were any deviations from the study protocol after approval was obtained please provide details of these changes in the Methods section of your manuscript.
Did this study involve local collaborators that are residents of the country where the research was conducted or members of the community studied? If you do not have any authors from said communities, please provide an explanation for this below.

Yes, this study was carried out by local collaborators, staff working on the Wellcome funded project ‘Tackling Histoplasmosis; a neglected disease impacting on equine health and human livelihoods’. These include veterinary research associates who are Ethiopian nationals and whose primary languages are regional languages including Oromic. The study was also organized in collaboration with regional NGOs SPANA and Brooke with support from the College of Veterinary Medicine and Agriculture, University of Addis Ababa.

Reported on page number: Not applicable, there was no devisation from the study protocol following approval.

Everyone listed as an author should meet PLOS’ criteria for authorship and all individuals who meet these criteria should be included in the author byline, rather than the acknowledgements. Authorship criteria is based on the International Committee of Medical Journal Editors (ICMJE) Uniform Requirements for Manuscripts Submitted to Biomedical Journals - for further information please see here: <https://journals.plos.org/plosone/s/authorship>.

**Human subjects research (e.g. health research, medical research, cross-cultural psychology)**

Did you obtain written informed consent from a representative of the local community or region before the research took place? How did you establish who speaks for the community? Details of written informed consent obtained from study participants should be reported separately in the Methods section of your manuscript.

Yes, prior to approaching potential participants for the study, regional representatives were consulted to provide consent to approach and seek participants. The initial meetings with regional representatives was supported by an introduction letter from the College of Veterinary Medicine and Agriculture. Written informed voluntary consent was sought from all participants in the study. Participants were provided with study information translated into Oromic or Amharic as appropriate, and were asked to provided written consent in either Oromic or Amharic. The study was introduced and carried out by Ethiopian nationals who translated all study questions into Oromic or Amharic. We aimed to speak with a wide variety of people with a range of different experiences, in order to represent diversity within the community, and participants were purposefully selected in this way.

How did members of the local community provide input on the aims of the research investigation, its methodology, and its anticipated outcome(s)?

The topic guide was created in response to experience of our NGO collaborators working with horse owners at NGO clinics in Ethiopia, and from previous findings from a participatory study involving equine owning community members in the same region (Scantlebury et al. 2015) and co-authored by Ethiopian nationals. For this study, the topic guide evolved once our lead author was based in Ethiopia. This was because this provided further opportunity to discuss with our Ethiopian colleagues about appropriate translations and what was most relevant for the context.

When engaging with the local community, how did you ensure that the informed consent documents and other materials could be understood by local stakeholders?

Our research team included Ethiopian nationals who translated all information and led all communication with local stakeholders. All study information sheets and consent forms were translated into Oromic and Amharic.

Will the findings of the research be made available in an understandable format to stakeholders in the community where the study was conducted (e.g. via a presentation, summary report, copies of publications, etc.)? Please provide details of how this will be achieved.

We have shared findings with our NGO collaborators who may adopt aspects of these findings into their outreach programmes with local communities. Additionally, we will prepare an summary leaflet to share with regional representatives to communicate study findings.

**Non-human subjects research using specimens/ animals collected as part of the study, or those housed in archival collections. Examples include archaeology, paleontology, botany and zoology.**

Did the permission you obtained from a local authority to perform the study include an agreement on access to outputs and benefit sharing? This may include procedures to enable fair distribution of the benefits and resources arising from the research performed. Please include any details of Prior Informed Consent and Benefit Sharing Agreements obtained. These may be required by field-specific regulations, for example the Convention on Biological Diversity (CBD) and the associated Nagoya Protocol.

Not applicable.

If the material used in your study was imported, please A) provide the year it was imported and B) indicate whether permits were obtained to import/export the materials used, C) provide details of any permits obtained. If this information is not available, please indicate this.

Not applicable

If you used archival specimens, please state how the material used in your study was acquired by the institute it is held in and provide details of any permits obtained for the original excavations/ sample collection. If this information is not available, please indicate this.

Not applicable

How was the potential cultural significance of the materials collected in your study to local communities considered in your research design? Were Indigenous peoples and/or local researchers and institutions involved with archaeological excavations / collection of specimens? If so, please provide a description of their involvement.

Not applicable

If your manuscript includes photographs of human remains please indicate whether authors obtained permission from descendants or affiliated cultural communities to do so.

Not applicable
