# Supplementary material for: Striving towards access to essential medicines for human and animal health; a situational analysis of access to and use of antifungal medications for histoplasmosis in Ethiopia
Source: PLoS One. 2023 Mar 9;18(3):e0278964. doi: 10.1371/journal.pone.0278964 (PMC9997978; doi:10.1371/journal.pone.0278964)
Supplement: S1 Table — (DOCX) [file pone.0278964.s002.docx]

Table of demographics of individuals contributing to interviews and focus groups *

| **Date** | **Region** | **Interview Number** | **Description** | | **Gender** | **# Years experience** | | **Language** |
| --- | --- | --- | --- | --- | --- | --- | --- | --- |
| 11/12/18 | Addis | 3 | Doctor - dermatologist | Male | | 10 years | English | |
| 11/12/18 | Addis | 4 | Doctor - dermatologist | Male | | 3 years | English | |
| 12/11/18 | Modjo | 9 | Doctor - GP | Male | | senior | English | |
| 13/11/18 | Bishoftu | 19 | Doctor - GP | Male | | 8 years | English | |
| 18/11/18 | Adama | 34 | Doctor - GP | Male | | 2 years | English | |
| 11/12/18 | Addis | 5 | Doctor - GP/internal med | Male | | Junior | English | |
| 15/11/18 | Holeta | 27 | Health Officer | Female | | 10 years | Amharic | |
| 12/11/18 | Modjo | 10 | Horse Owner | Male | | 3 years | Amharic | |
| 13/11/18 | Bishoftu | 12 | Horse Owner | Male | | 6 months/2 year | Amharic | |
| 13/11/18 | Bishoftu | 12 | Horse Owner | Male | | 2 years | Amharic | |
| 13/11/18 | Bishoftu | 12 | Horse Owner | Male | | 8 months/ 3 years | Amharic | |
| 13/11/18 | Bishoftu | 12 | Horse Owner | Male | | 4 months | Amharic | |
| 13/11/18 | Bishoftu | 13 | Horse Owner | Male | | 1 year | Oromic | |
| 13/11/18 | Bishoftu | 13 | Horse Owner | Male | | 2 months | Oromic | |
| 13/11/18 | Bishoftu | 13 | Horse Owner | Male | | 2 years | Oromic | |
| 13/11/18 | Bishoftu | 13 | Horse Owner | Male | | 1 year | Oromic | |
| 13/11/18 | Bishoftu | 13 | Horse Owner | Male | | 1 year | Oromic | |
| 13/11/18 | Bishoftu | 13 | Horse Owner | Male | | 1 year | Oromic | |
| 15/11/18 | Holeta | 22 | Horse Owner | Male | |  | Oromic | |
| 15/11/18 | Holeta | 22 | Horse Owner | Male | |  | Oromic | |
| 15/11/18 | Holeta | 22 | Horse Owner | Male | |  | Oromic | |
| 15/11/18 | Holeta | 23 | Horse Owner | Male | |  | Oromic | |
| 15/11/18 | Holeta | 23 | Horse Owner | Male | |  | Oromic | |
| 15/11/18 | Holeta | 23 | Horse Owner | Male | |  | Oromic | |
| 18/11/18 | Adama | 29 | Horse Owner | Male | | 1 year | Oromic | |
| 18/11/18 | Adama | 29 | Horse Owner | Male | | 1 year | Oromic | |
| 18/11/18 | Adama | 29 | Horse Owner | Male | | 7 years | Oromic | |
| 18/11/18 | Adama | 29 | Horse Owner | Male | | 2 years | Oromic | |
| 18/11/18 | Adama | 29 | Horse Owner | Male | | 3 years | Oromic | |
| 18/11/18 | Adama | 30 | Horse Owner | Male | | 1 year | Oromic | |
| 18/11/18 | Adama | 30 | Horse Owner | Male | | 1 year | Oromic | |
| 18/11/18 | Adama | 30 | Horse Owner | Male | | 6 months | Oromic | |
| 18/11/18 | Adama | 30 | Horse Owner | Male | | 8 months | Oromic | |
| 18/11/18 | Adama | 31 | Horse Owner | Male | | 2 years | Amharic | |
| 18/11/18 | Adama | 31 | Horse Owner | Male | | 2 years | Amharic | |
| 18/11/18 | Adama | 31 | Horse Owner | Male | | 1 year | Amharic | |
| 20/11/18 | Bishoftu | 37 | Horse Owner | Male | | 2 years | Oromic | |
| 20/11/18 | Bishoftu | 37 | Horse Owner | Male | | 2 years | Oromic | |
| 20/11/18 | Bishoftu | 38 | Horse Owner | Male | | 8 months | Oromic | |
| 20/11/18 | Bishoftu | 38 | Horse Owner | Male | | 3 years | Oromic | |
| 20/11/18 | Bishoftu | 38 | Horse Owner | Male | | 1 year | Oromic | |
| 20/11/18 | Bishoftu | 38 | Horse Owner | Male | | 2 years | Oromic | |
| 20/11/18 | Akaki | 41 | Horse Owner | Male | | 7 years | Amharic | |
| 20/11/18 | Akaki | 41 | Horse Owner | Male | | 2 years | Amharic | |
| 20/11/18 | Akaki | 41 | Horse Owner | Male | | 2 years | Amharic | |
| 20/11/18 | Akaki | 41 | Horse Owner | Male | | 1 year | Amharic | |
| 20/11/18 | Akaki | 42 | Horse Owner | Male | | 5 years | Oromic | |
| 20/11/18 | Akaki | 42 | Horse Owner | Male | | 2 years | Oromic | |
| 20/11/18 | Akaki | 42 | Horse Owner | Male | | 3 years | Oromic | |
| 20/11/18 | Akaki | 42 | Horse Owner | Male | | 1 year | Oromic | |
| 13/11/18 | Bishoftu | 17 | ParaVet | Male | | 11 years | Amharic | |
| 13/11/18 | Bishoftu | 17 | ParaVet | Male | | 16 years | Amharic | |
| 11/12/18 | Addis | 1 | Pharmacist | Male | | senior | English | |
| 11/12/18 | Addis | 2 | Pharmacist | Female | | junior | English | |
| 11/12/18 | Addis | 6 | Pharmacist | Male | | Senior | Amharic | |
| 12/11/18 | Modjo | 8 | Pharmacist | Male | | senior | English | |
| 13/11/18 | Bishoftu | 20 | Pharmacist | Male | | 2 years | Amharic | |
| 14/11/18 | Bishoftu | 21 | Pharmacist | Female | | 3 years | Amharic | |
| 15/11/18 | Holeta | 28 | Pharmacist | Male | | 5 years | Oromic | |
| 18/11/18 | Adama | 33 | Pharmacist | Male | | 11 years | English | |
| 18/11/18 | Adama | 33 | Pharmacist | Male | | 25 years | English | |
| 19/11/18 | Addis | 35 | Pharmacist | Male | | 3 years | Amharic | |
| 19/11/18 | Addis | 36 | Pharmacist | Male | | 8 years | Amharic | |
| 20/11/18 | Bishoftu | 39 | Pharmacist | Male | | 3 years | English | |
| 12/11/18 | Modjo | 11 | Pharmacist | Female | | 20 years | Amharic | |
| 13/11/18 | Bishoftu | 18 | Pharmacist | Male | | 3 years | English | |
| 12/11/18 | Modjo | 7 | Vet | Male | | 3 years | English | |
| 12/11/18 | Modjo | 7 | Vet | Male | | 3 years | English | |
| 13/11/18 | Bishoftu | 16 | Vet | Male | | 5 years | English | |
| 18/11/18 | Adama | 32 | Vet | Male | | 3 years | English | |
| 18/11/18 | Adama | 32 | Vet | Male | | 5 years | English | |
| 18/11/18 | Adama | 32 | Vet | Male | | 30 years | English | |
| 18/11/18 | Adama | 32 | Vet | Male | | 4 months | English | |
| 20/11/18 | Akaki | 40 | Vet | Male | | 20 years | Amharic | |
| 15/11/18 | Holeta | 24 | Vet Assist / Paravet | Male | | 12 years | Oromic | |
| 15/11/18 | Holeta | 25 | Vet Assist / Paravet | Male | | 1 year | Oromic | |
| 13/11/18 | Bishoftu | 14 | Vet Student | Male | | final year | English | |
| 13/11/18 | Bishoftu | 15 | Vet Student | Male | | final year | English | |
| 15/11/18 | Holeta | 26 | Vet Student | Male | | final year | English/Oromic | |

*Focus groups are indicated by having the same interview number including multiple individuals.
