# Supplementary material for: Striving towards access to essential medicines for human and animal health; a situational analysis of access to and use of antifungal medications for histoplasmosis in Ethiopia
Source: PLoS One. 2023 Mar 9;18(3):e0278964. doi: 10.1371/journal.pone.0278964 (PMC9997978; doi:10.1371/journal.pone.0278964)
Supplement: S1 File — Key questions and topic areas for equid owners, veterinary surgeons, medics and pharmacists. (DOCX) [file pone.0278964.s003.docx]

**Equid Owner Focus Group Discussion Guide**

Hello, my name is Eleanor Robertson, I am a final year veterinary student and a veterinary researcher from the University of Liverpool. These are my colleagues, Dr. Kelly Wood a veterinary surgeon from the University of Liverpool, and veterinary surgeons, Dr. Cherinet Abera from Brooke and Dr. Kabeba Deressa from SPANA Ethiopia. These are non-governmental charities that assist with equine health and welfare in Ethiopia.

I would like to ask you about your understanding and opinions on types of medications and treatments for fungal infections. I am interested in your opinions, experiences and thoughts about use of these medicines and how you can access them. This group discussion should take no longer than 60 minutes and you are free to leave at any time. You are free to choose whether or not you wish to talk with me today. We will record the session so that we can review the discussion later on for general analysis. However, any comments that you make will be kept anonymous and the information will not be linked back to an individual. We will keep the data safely secured and will explore what you say compared to other groups to gain a big picture understanding of how anti-fungal medicines are used at the moment and to describe some of the challenges with their use here.

Nothing that personally identifies you will be used in the study to ensure you remain anonymous. The responses will be combined and written up into a report that will be shared to veterinary surgeons who deal with fungal infections, and potentially with policy makers that decide about access to anti-fungals. If you have any questions now or in the future about this study please feel free to ask or later on you can contact me using the details provided on the participant information sheet.

1. **Before we begin the discussion, does anyone have any questions?**
2. **Please take a look at this picture and tell me what you think.**

(show pictures A, B, C, D, E, F)

- 1. Have you seen horses with this? What is it known as?
  2. What do you think causes this in horses? / How do you think this condition develops in horses?
  3. What do you understand by the term ‘fungal infection’? What is transmitted between horses to spread the infection?

1. **What would you do if you owned a horse that looked like this (pictures)?**
   1. Does this condition require any treatment?
   2. If yes, how would you treat a condition such as this?
   3. Who would you seek advice from about treating this (picture)?
   4. IF A LIST – which would you attempt first and why?
2. **Could you tell me more about the process of treating this condition?**
   1. Is it difficult or easy to provide this treatment?
   2. Where do you obtain treatment from? - Prescription?
   3. Is it expensive or cheap to obtain this treatment? how long is it needed for? (ongoing costs?)
   4. What do you think influences your choice of treatment? *E.g. friends/professional advice/ personal experience/access/ advertising/price*
   5. Do you think the treatment works? If not, why do you think that might be? Why do you think these problems exist?
   6. Do you have any concerns about the safety of this treatment? Have you seen animals with bad effects after treatment??
3. **Are there any risks to humans with handling animals with this condition?**
   1. Do you think this condition is a problem for people?
   2. What about with the administration of treatment, are there any dangers?
4. **Please take a look at this picture and tell me what you think (G,H,I,J)**
   1. Have you seen people with these conditions? What is it known as?
   2. Is this a common problem here in Ethiopia?
   3. What do you think causes this in people? / How do you think this condition develops?
   4. What would you do if a family member had this? (Probe – would you seek treatment? If so where? Or what would you treat with?)
5. **Closing Questions**
   1. Is there anything else you want to tell me or clarify?
   2. Do you have any questions for me?

Thank you very much for taking the time to talk to me today. I really appreciate your participation in the study; you input was very useful for my research. If you have any questions later, my contact details are on the participation information form. Please do not hesitate to get in touch at any time.

**Vet Interview Guide**

Hello, my name is Eleanor Robertson, I am a final year veterinary student and a veterinary researcher from the University of Liverpool. These are my colleagues, Dr. Kelly Wood a veterinary surgeon from the University of Liverpool, and veterinary surgeons, Dr.Cherinet Abera from Brooke and Dr.Kabeba Deressa from SPANA Ethiopia. These are non-governmental charities that assist with equine health and welfare in Ethiopia.

I would like to ask you about your understanding and opinions on types of medications and treatments for fungal infections. I am interested in your opinions, experiences and thoughts about use of these medicines and how you can access them. This group discussion should take no longer than 60 minutes and you are free to leave at any time. You are free to choose whether or not you wish to talk with me today. We will record the session so that we can review the discussion later on for general analysis. However, any comments that you make will be kept anonymous and the information will not be linked back to an individual. We will keep the data safely secured and will explore what you say compared to other groups to gain a big picture understanding of how anti-fungal medicines are used at the moment and to describe some of the challenges with their use here.

Nothing that personally identifies you will be used in the study to ensure you remain anonymous. The responses will be combined and written up into a report that will be shared to veterinary surgeons who deal with fungal infections, and potentially with policy makers that decide about access to anti-fungals. If you have any questions now or in the future about this study please feel free to ask or later on you can contact me using the details provided on the participant information sheet.

1. **Before we begin the discussion, does anyone have any questions?**
2. **Please take a look at this picture and tell me what you think (A,B,C,D,E,F)**
   1. Have you seen horses with this? What is it known as?
   2. Is this a common problem here in Ethiopia?
   3. What do you think causes this in horses? / How do you think this condition develops in horses?
   4. Do you think this is a problem for horses?
3. **What would you do if you saw a horse that looked like this (pictures)?**
   1. Does this condition require any treatment?
   2. How do you treat a condition such as this?
   3. IF A LIST – which would you attempt first and why?
4. **Could you tell me more about the process of treating this condition?**
   1. Where do you obtain treatment from?
   2. Is it easy or difficult to obtain treatment?
   3. Is it expensive or cheap to obtain this treatment?
   4. how long is it needed for? (ongoing costs?)
   5. What do you think influences your recommendation of treatment? *E.g. previous cases/colleagues/access/ advertising/price*
   6. Any challenges with treatment? Do you get a good response to treatment? If not, why do you think that might be e.g. compliance/ resistance? Why do you think these problems exist?
   7. Do you have any concerns about the safety of this treatment? Have you seen horses or donkeys with adverse effects after treatment??
5. **Are there any risks to humans with handling animals with this condition?**
   1. Do you think this condition is a problem for people?
   2. What about with the administration of treatment, are there any dangers?
   3. Do you know of any anti-fungal use in other situations? e.g. agricultural use/ to reduce disease in crops / flowers?
6. **Please take a look at this picture and tell me what you think (G,H,I,J)**
   1. Have you seen people with these conditions? What is it known as?
   2. Is this a common problem here in Ethiopia?
   3. What do you think causes this in people? / How do you think this condition develops?
   4. What would you do if a family member had this? (Probe – would you seek treatment? If so where? Or what would you treat with?)
7. **Closing Questions**
   1. Is there anything else you want to tell me or clarify?
   2. Do you have any questions for me?

Thank you very much for taking the time to talk to me today. I really appreciate your participation in the study; you input was very useful for my research. If you have any questions later, my contact details are on the participation information form. Please do not hesitate to get in touch at any time.

**Medic Interview Guide**

Hello, my name is Eleanor Robertson, I am a final year veterinary student and a veterinary researcher from the University of Liverpool. These are my colleagues, Dr. Kelly Wood a veterinary surgeon from the University of Liverpool, and veterinary surgeons, Dr. Cherinet Abera from Brooke and Dr. Kabeba Deressa from SPANA Ethiopia. These are non-governmental charities that assist with equine health and welfare in Ethiopia.

I would like to ask you about your understanding and opinions on types of medications and treatments for fungal infections. I am interested in your opinions, experiences and thoughts about use of these medicines and how you can access them. This group discussion should take no longer than 60 minutes and you are free to leave at any time. You are free to choose whether or not you wish to talk with me today. We will record the session so that we can review the discussion later on for general analysis. However, any comments that you make will be kept anonymous and the information will not be linked back to an individual. We will keep the data safely secured and will explore what you say compared to other groups to gain a big picture understanding of how anti-fungal medicines are used at the moment and to describe some of the challenges with their use here.

Nothing that personally identifies you will be used in the study to ensure you remain anonymous. The responses will be combined and written up into a report that will be shared to veterinary surgeons who deal with fungal infections, and potentially with policy makers that decide about access to anti-fungals. If you have any questions now or in the future about this study please feel free to ask or later on you can contact me using the details provided on the participant information sheet.

1. **Before we begin the discussion, does anyone have any questions?**
2. **Please take a look at this picture and tell me what you think.**

(show pictures G, H, I, J depicting humans with fungal infection)

- 1. Have you seen people with this? What is it known as?
  2. Are these common here in Ethiopia?
  3. What do you think causes this in people? / How do you think this condition develops in people? (note which picture is discussed)
  4. Do you think these types of conditions are a problem for people?
  5. What are your experiences of treating fungal infections here?

1. **What would you do if you saw a patient that presented like this (case presentation)?**
   1. What would you think in this case? Differentials? (Probe: This could be a case of histoplasmosis, have you had any experience with this disease?)
   2. Does this condition require any treatment?
   3. If yes, how do you treat a condition such as this (picture)?
   4. IF A LIST – which would you attempt first and why?
2. **Could you tell me more about the process of treating histoplasmosis?**
   1. Is it difficult or easy to provide this treatment?
   2. Where do you / your patients obtain treatment from? - Prescription?
   3. Is it expensive or cheap to obtain this treatment? How long is it needed for? (ongoing costs?)
   4. What do you think influences your recommendation of treatment? *E.g. previous cases/colleagues/access/ advertising/price*
   5. Any challenges with treatment? Do you get a good response to treatment? If not, why do you think that might be e.g. compliance/ resistance? Why do you think these problems exist?
   6. Do you have any concerns about the safety of this treatment? Have you seen patients with adverse effects after treatment??
3. **We are just going to show you some pictures of animals because we want to know if you think there any risks to humans with handling animals with any of these conditions.** (Show photos A, B, C, D, E, F) (Probe: possible treatments for these are Fluconazole, Amphotericin-b, Potassium Iodide).
   1. What about with the administration of treatment, are there any dangers?
   2. Are humans in contact with this or similar drugs from other sources that you know of e.g. environmental or agricultural use?
   3. Causative agents – what are they? – is this a risk to people?
4. **Closing Questions**
   1. Is there anything else you want to tell me or clarify?
   2. Do you have any questions for me?

Thank you very much for taking the time to talk to me today. I really appreciate your participation in the study; you input was very useful for my research. If you have any questions later, my contact details are on the participation information form. Please do not hesitate to get in touch at any time.

**Pharmacist Interview Guide**

Hello, my name is Eleanor Robertson, I am a final year veterinary student and a veterinary researcher from the University of Liverpool. These are my colleagues, Dr. Kelly Wood a veterinary surgeon from the University of Liverpool, and veterinary surgeons, Dr. Cherinet Abera from Brooke and Dr. Kabeba Deressa from SPANA Ethiopia. These are non-governmental charities that assist with equine health and welfare in Ethiopia

I would like to invite you to take part in a short discussion for a research project that is exploring the use of medicines against fungal disease in Ethiopia. If you are willing, I would like to ask you about your understanding and opinions on availability and types of medications and treatments for fungal infections. I am interested in your opinions, experiences and thoughts about use of these medicines and how you can access them. You are free to choose whether or not you wish to talk with me today. With your permission, we will record the session so that we can review the discussion later on for general analysis. However, any comments that you make will be kept anonymous meaning that we will not identify any individual in any reports or analysis of what you discuss, and the information will not be linked back to an individual. This group discussion should take no longer than 60 minutes and you are free to leave at any time. We will keep the data safely secured and will explore what you say compared to other groups to gain a big picture understanding of how anti-fungal medicines are used at the moment and to describe some of the challenges with their use here.

Nothing that personally identifies you will be used in the study to ensure you remain anonymous. The responses will be combined and written up into a report that will be shared to veterinary surgeons who deal with fungal infections, and potentially with policy makers that decide about access to anti-fungals. If you have any questions now or in the future about this study please feel free to ask or later on you can contact me using the details provided on the participant information sheet.

1. **Before we begin the discussion, does anyone have any questions?**
2. **Please take a look at these pictures (G,I,H,J) and tell me what you think.**
   1. Have you seen people or animals with this? What is it known as?
   2. Are these a common problem here in Ethiopia?
   3. What do you think causes this? / How do you think this condition develops in people or horses?
   4. Do you think this is a problem for people?
   5. What do you understand by the term ‘fungal infection’? What is transmitted between people to spread the infection?
3. **What would you do if you saw a client that presented like this (pictures)?**
   1. Do you commonly provide treatment for animals as well as people?
   2. Does this condition require any treatment?
   3. If yes, how do you treat a condition such as this (picture) or where would you suggest your client go for advice?
   4. IF A LIST – which would you attempt first and why?
4. **Could you tell me more about the process of treating this condition?**
   1. Is it difficult or easy to provide this treatment? - Prescription?
   2. Is it expensive or cheap to obtain this treatment? how long is it needed for? (ongoing costs?)
   3. What do you think influences your recommendation of treatment? *E.g. previous cases/colleagues/access/ advertising/price*
   4. Do you have any concerns about the safety of this treatment? Have you seen clients with adverse effects after treatment??
   5. Any challenges with treatment? Do you get a good response to treatment? If not why do you think that might be e.g. compliance/ resistance? Why do you think these problems exist?
5. **Are there any risks to humans with handling animals with any of these conditions?** (Show photos A, B,C,D,E,F)
   1. Do you think this condition is a problem for people?
   2. What about with the administration of treatment, are there any dangers?
   3. Are humans in contact with this or similar drugs from other sources e.g. agricultural use?
6. **Closing Questions**
   1. Is there anything else you want to tell me or clarify?
   2. Do you have any questions for me?

Thank you very much for taking the time to talk to me today. I really appreciate your participation in the study; you input was very useful for my research. If you have any questions later, my contact details are on the participation information form. Please do not hesitate to get in touch at any time.
