# Supplementary material for: Striving towards access to essential medicines for human and animal health; a situational analysis of access to and use of antifungal medications for histoplasmosis in Ethiopia
Source: PLoS One. 2023 Mar 9;18(3):e0278964. doi: 10.1371/journal.pone.0278964 (PMC9997978; doi:10.1371/journal.pone.0278964)
Supplement: S2 File — (DOCX) [file pone.0278964.s004.docx]

A 25‐year‐old woman presents with fever (38.7°C) and flu‐like symptoms. The patient has a 3-week history of a dry cough, chest pain, and shortness of breath on exertion. She is HIV+ and has been experiencing night sweats for the past week.

Physical examination was normal, oxygen saturation was 93% on air and a chest X‐ray showed diffuse bilateral miliary nodules [34].

Sputum was negative on staining for acid‐fast bacilli and TB culture negative. Further, a Broncho-alveolar lavage specimen was culture negative for TB. What would be your next steps with this patient?
